# *indexGIXS*: software for visualizing and interactive indexing of grazing-incidence scattering data

**DOI:** 10.1107/S1600576726002608

**Published:** 2026-05-14

**Authors:** Detlef-M. Smilgies, Ruipeng Li

**Affiliations:** a Center for Advanced Microelectronics Manufacturing (CAMM) and Materials Science and Engineering Program Binghamton University Binghamton NY13902 USA; b R. F. Smith School of Chemical and Biomolecular Engineering Cornell University Ithaca NY14853 USA; c National Synchrotron Light Source II Brookhaven National Laboratory Upton NY11973 USA; Universität Duisburg-Essen, Germany

**Keywords:** grazing-incidence scattering methods, indexing, nanostructures, organic thin films, computer programs, *indexGIXS*

## Abstract

Grazing-incidence small- and wide-angle X-ray scattering are widely applied for the study of functional organic thin films, be it for the characterization of nanostructured morphologies in block copolymers, nanocomposites or nanoparticle assemblies, or the packing and orientation of organic semiconductors such as small aromatic molecules and conjugated polymers. Typically, organic thin films are uniaxial powders, with specific crystallographic planes oriented parallel to the substrate surface. The associated fiber-texture scattering patterns are complicated by refraction corrections and multiple scattering. Here, we present an interactive graphics tool to index such patterns.

## Abstract 2

A program is described for visualizing grazing-incidence scattering data and interactive indexing of the observed diffraction spots.

## 1. Introduction

With the popular use of grazing-incidence X-ray scattering techniques for the characterization of thin films in nanoscience and organic electronics there is an increasing demand for graphical software tools in order to index scattering patterns interactively (Tate *et al.*, 2006 ▸; Breiby *et al.*, 2008 ▸; Jiang, 2015 ▸). Moreover, such a program should run on a flexible and low-cost platform that can be installed conveniently under the most common operating systems. The free and open-source *Scilab* software package (https://www.scilab.org) provides such a platform: it features a high-level programming language for easy manipulation of arrays and images, as well as powerful graphics and an easy-to-use graphical user interface. In the following we describe the interactive *indexGIXS* program for data visualization and indexing, which is based on our earlier work on the indexing problem (Smilgies *et al.*, 2005 ▸; Smilgies & Blasini, 2007 ▸).

## 2. Program description

### 2.1. Detector space versus reciprocal space

In order to keep processing time at a minimum, it is efficient to calculate positions of diffraction spots in detector space (pixels for area detectors, angles for reciprocal-space maps), rather than to perform the non-linear transformation of all pixels in the raw data to reciprocal space. This issue is most pronounced for grazing-incidence wide-angle X-ray scattering (GIWAXS); in grazing-incidence small-angle X-ray scattering (GISAXS), *q* space is essentially a linear function of the scattering angles, and reciprocal-space maps may be generated in good approximation by a linear transformation.

Even in the realm of small-angle scattering, there is still an advantage of remaining in detector space, because X-ray refraction, reflection, and dynamic effects are pronounced at small scattering angles close to the critical angle of the film (Lee *et al.*, 2005 ▸; Busch *et al.*, 2006 ▸; Tate *et al.*, 2006 ▸; Stein *et al.*, 2007 ▸; Breiby *et al.*, 2008 ▸; Jiang *et al.*, 2011 ▸; Smilgies, 2022 ▸). After all, of interest is the scattering vector inside the film. Refraction and reflection corrections are essential for the interpretation of GISAXS data. These conditions are automatically detected in *indexGIXS* and diffraction-spot positions are corrected accordingly. Finally there are the diffuse-scattering intensities close to the beamstop, where Bragg’s law cannot be fulfilled and the Ewald sphere only cuts through the diffuse halo of the proper Bragg reflections (Smilgies, 2019 ▸). With all these complications, we found it useful to simulate scattering patterns in detector space rather than convert detector images to *q* space and then face the problem of detangling the various scattering processes.

### 2.2. Area-detector data display

The *indexGIXS* program reserves a large part of its application window for the detailed display of the measured scattering patterns. All types of area detectors in use at the Cornell High Energy Synchrotron Source are supported (MedOptics, FLIcam, Dectris Pilatus 100K, 200K and 300K, GE Typhoon 7000 image plate reader, Eiger 1M). It is also possible to read in data from G2 reciprocal-space maps employing a linear diode array in conjunction with Soller slits, which are mounted on a two-axis detector arm (Smilgies *et al.*, 2005 ▸; Nowak *et al.*, 2006 ▸). By user request, additional data formats were added, such as for Pilatus 1M and 2M detectors at the Advanced Light Source, the Australian Synchrotron, and NSLS-II beamlines. In addition, the EDF format for laboratory-based instruments was recently added. The specifics of the chosen detector, such as pixel size and number, TIFF data format, and typical intensity range, can be displayed in the *det_info* menu by clicking on the *detector type* button. Other detectors using the TIFF format can be added directly by choosing the type *custom*. Due to hardware and software limitations, a maximum image size of 10 MB should not be exceeded for unbinned images; for larger detectors, binning should be used. A fast binning mode for 2 × 2, 3 × 3 *etc*. pixels is provided and can be set up in the *detector type* menu.

**Figure 1:**
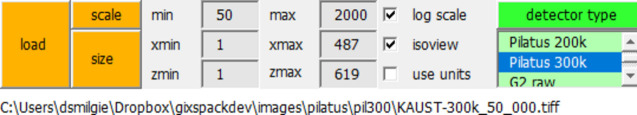
Data-file input and manipulation menu. The *detector type* pull-down menu provides a list of supported detectors. The *detector type* pushbutton calls a menu that displays the default settings for the supported detectors or permits the user to define a custom detector. When a detector type is chosen in the list box below, images can be loaded (*load* pushbutton). The image file name is displayed below the menu. The *scale* and *size* pushbuttons in conjunction with the adjacent input boxes permit the user to fine-tune the display parameters (see the main text).

When a detector is chosen, data can be read using the *load* button (see Fig. 1 ▸). The display of data can be further refined by specifying intensity range (input boxes and *scale* button) and image size (input boxes and *size* button). Zoom and pan operations can also be performed using built-in *Scilab* graphics features with the mouse wheel or by dragging a point inside the image, respectively. However, for comparing between specific parts of different detector images, the *size* button remains convenient.

Presets for the intensity range corresponding to the detector type are preloaded when reading the image. There is a choice of linear versus logarithmic display (*log scale*) as well as for providing an appropriate aspect ratio for rectangular detectors such as the Dectris Pilatus detectors (*isoview*); alternatively, the plot is maximized on the available area if *isoview* is unchecked. Image units are given in pixels or scattering-vector magnitude *q* for area detectors, or in degrees for reciprocal-space maps (*use units*). The *q* transformation is limited to the small-angle approximation and is meant for publication purposes, while indexing should be primarily done in pixel space.

### 2.3. Scattering-pattern simulation

The parameter set for specifying a grazing-incidence X-ray scattering pattern is quite elaborate and the menu, divided into logical submenus, is displayed to the right of the data. When the real-space lattice parameters are provided, the reciprocal lattice parameters are determined, and the three-dimensional reciprocal lattice vectors are calculated (Smilgies *et al.*, 2005 ▸; Smilgies & Blasini, 2007 ▸).

**Figure 2:**
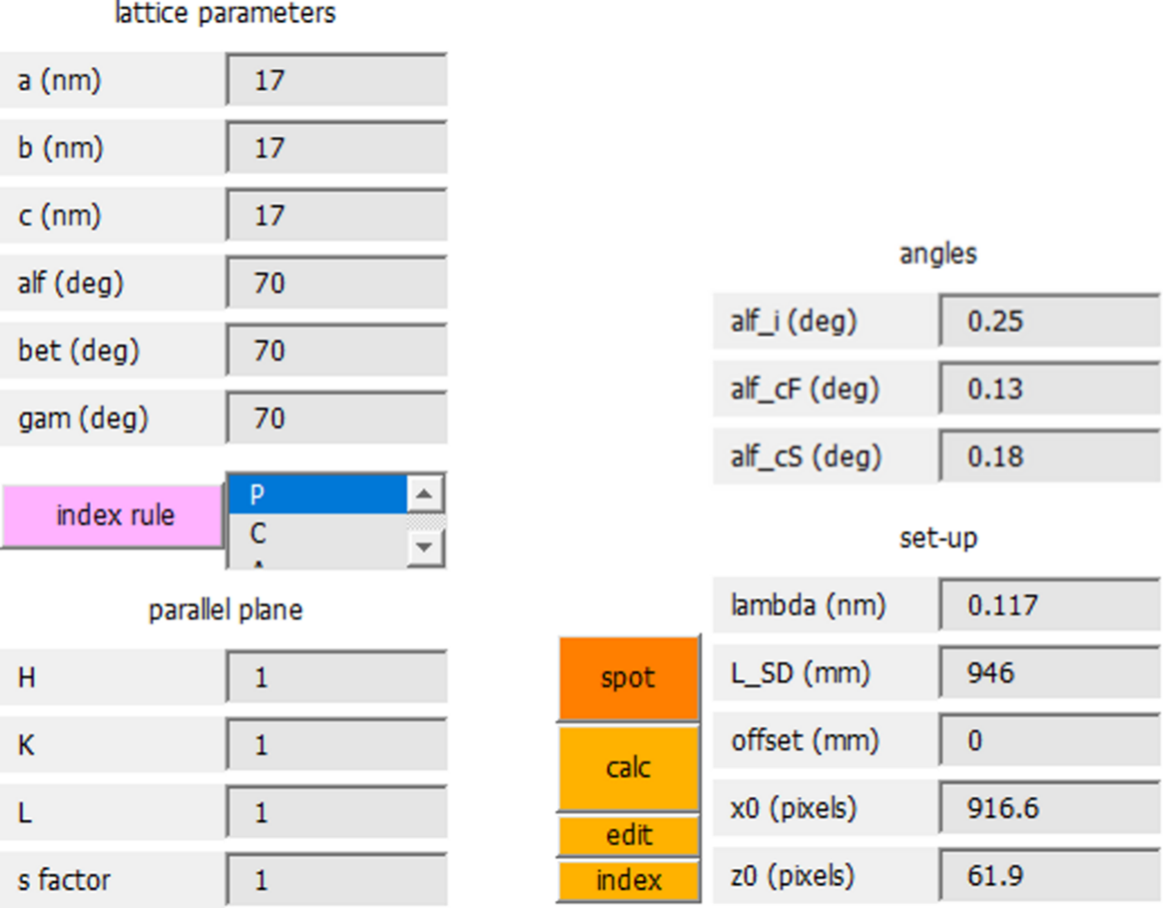
Input of lattice and setup parameters. For convenience, all relevant parameters are displayed in the right sidebar. On the left of the figure, the top part of the sidebar with lattice parameters (*a, b, c, alf, bet, gam*), *index rule* pull-down menu, indices of the texture plane (*H, K, L*) and the *s factor* is shown. On the right, the lower part of the sidebar with angle menu and setup menu is shown. The *calc* pushbutton (orange) initializes the calculation. Numerical output can be inspected, modified or saved using *edit*. The pushbutton *index* displays diffraction indices in the diffraction image. The *spot* pushbutton opens a menu for customizing the plot, *i.e.* to select spot symbol, color and rim color, as well as the line style for the horizon and critical angles.

The index rules pertaining to the space group can be put in directly using the Bravais lattice types [primitive (*P*), centered (*C*), body centered (*I*) or face centered (*F*)]; additional screw axes and glide planes may be specified in the space-group menu accessed by clicking on the *index rule* pushbutton (see Fig. 2 ▸), which is useful for small-molecule lattices covering the triclinic, monoclinic and orthorhombic systems. The *index rule* menu also permits the user to set limits for the maximum *HKL* values used in the calculation, as well as to choose whether reflections are calculated for the left (*L*), right (*R*) or both (*B*) sides of the incident plane. Common high-symmetry lattices such as simple cubic (*sc*), face-centered cubic (*fcc*), body-centered cubic (*bcc*), hexagonal close-packed (*hcp*) and diamond (*dia*), as well as common block-copolymer structures such as single and double gyroid (*sgl gyr, dbl gyr*), hexagonal cylinders (*cyl*), and lamellae (*lam*), are generated automatically, if the *a* lattice parameter is specified. The predefined lattice types are most useful when it is clear that simple lattices were formed, in particular for block copolymers (Paik *et al.*, 2010 ▸; Chavis *et al.*, 2015 ▸), mesoporous structures and nanocomposites (Crossland *et al.*, 2009 ▸), as well as nanocrystal superlattices (Bian *et al.*, 2011 ▸; Zhang *et al.*, 2012 ▸; Weidman *et al.*, 2016 ▸).

A typical feature of thin films is that there is a preferential lattice plane formed parallel to the substrate surface. The Miller indices (*H, K, L*) of this crystallographic plane have to be specified explicitly. For the pre-defined high-symmetry lattices the close-packed planes are assumed to form parallel to the substrate. Calculations are only performed when hitting the *calc* pushbutton; hence, even when starting with a high-symmetry structure, lattice parameters and parallel planes can still be further modified before the calculation. With given lattice parameters and the parallel-plane indices, the reciprocal lattice vectors corresponding to this specific texture are generated and the associated diffraction-spot locations are superimposed on the detector image.

A special role is played by the *s factor*, where *s* stands for both swelling and shrinking. For thin films processed from solution or annealed in solvent vapor, the lattice spacings perpendicular to the substrate tend to expand during exposure to solvent vapor or shrink during drying, often past the equilibrium high-symmetry lattices. This non-equilibrium effect is pronounced in block copolymers with up to 50% swelling or shrinkage [see, for instance, Crossland *et al.* (2009 ▸) and Paik *et al.* (2010 ▸)], but occurs also to a lesser degree in nanoparticle assemblies (2–5%). While such lattices display a lower symmetry in a crystallographic sense, it is still useful to compare them with the high-symmetry equilibrium phase. In particular, it is easier to find an indexing when starting from a high-symmetry phase with a close-packed plane parallel to the substrate and then to adjust the *s factor*.

Other input parameters include the relevant angles such as incident angle (*alf_i*) and the critical angles of the substrate (*alf_cS*) and thin film (*alf_cF*). Finally, the experimental parameters, such as the X-ray wavelength (*lambda*) and the sample-to-detector distance (*L_SD*), can be specified. Furthermore, the pixel position of the direct beam on the detector (*x0*, *z0*) needs to be provided. The *offset* parameter is used when the detector is shifted horizontally from the position where the direct-beam position was measured originally. This parameter can be of importance when working with a small detector (such as a Pilatus 100K), when the detector needs to be shifted horizontally to collect diffraction spots at higher scattering angles.

## 3. Use of the program

### 3.1. Calibration of the setup

The first information to obtain for a given setup is the wavelength of the X-ray beam and, in the case of small- or wide-angle scattering, the sample–detector distance *L_SD*; the latter is usually determined with a standard, for example, silver behenate powder for small-angle scattering and ceria powder for wide-angle scattering. Finally, the *x0* and *z0* positions of the direct beam on the detector need to be determined in pixel numbers, taken with an appropriate attenuator.

### 3.2. Refraction parameters

**Figure 3:**
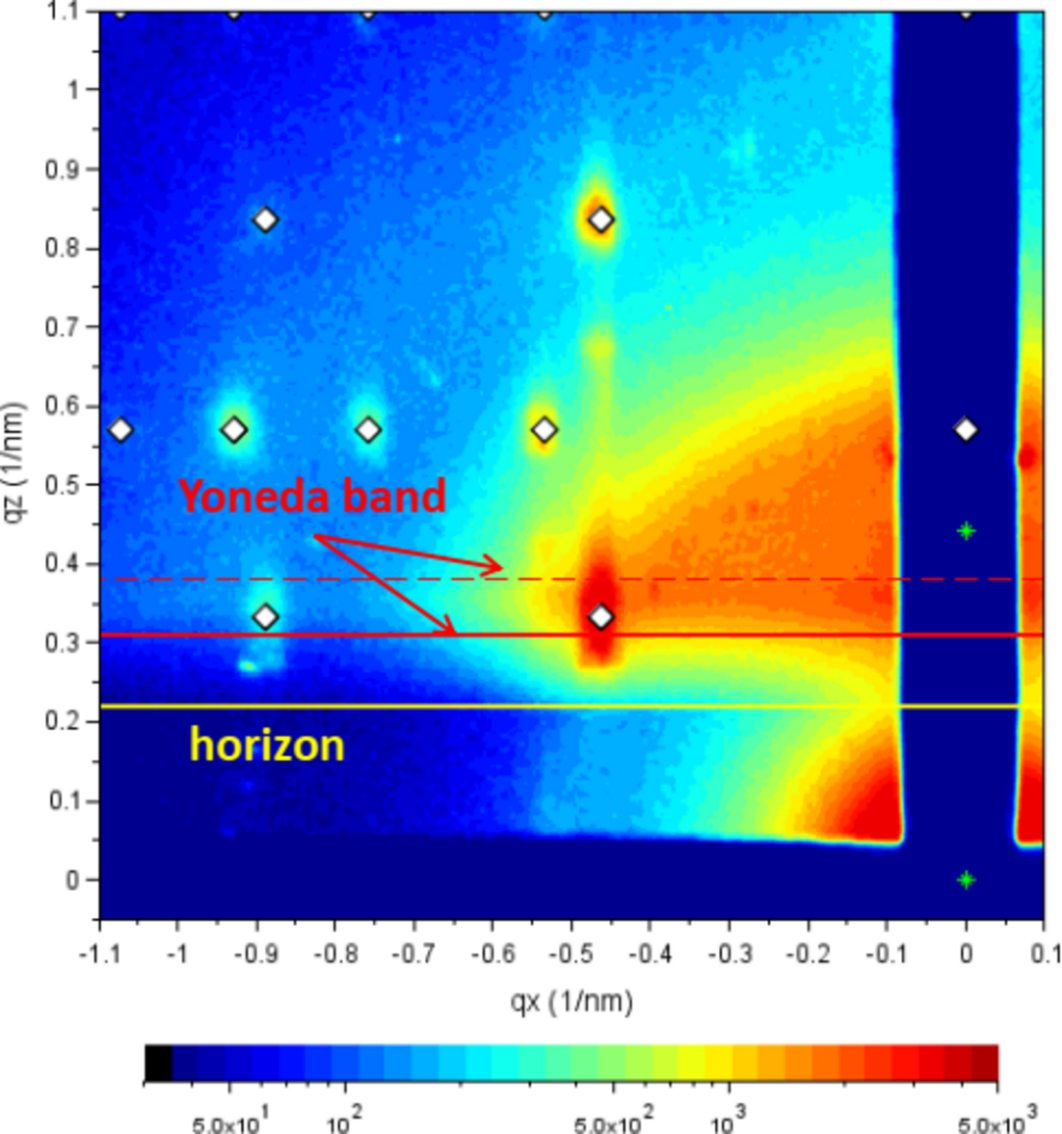
Revision of refraction parameters: direct and reflected beams are indicated by stars (green). Often tails of the reflected beam can be discerned close to the beamstop. A small increase in background scattering intensity indicates the position of the horizon (solid yellow line). Both reflected-beam tails and horizon are solely dependent on the incident angle. The Yoneda band, the band of enhanced scattering intensity between the critical angles of the film and the substrate (Smilgies, 2022 ▸), helps to revise the optical parameters. The film Yoneda line is found at the first maximum of the scattering intensity coming from the horizon (solid red line). The substrate Yoneda line appears often only as a shoulder (dashed red line). Usually a calculation from the substrate optical constants (CXRO, 2021 ▸) results in a good fit. Reproduced from Smilgies (2025 ▸) under the terms of the Creative Commons Attribution (CC BY) license (https://creativecommons.org/licenses/by/4.0/).

Typically, the incident angle of the X-ray beam is determined during sample lineup. The critical angles of the film and the substrate can be measured if lineup is combined with a reflectivity measurement covering the incident-angle range from 0 to at least twice *alf_cS*. The substrate critical angle can also be calculated from the known optical constants of the substrate. During *in situ* experiments such as solvent-vapor processing (Posselt *et al.*, 2017 ▸), it can happen that the sample moves by solvent collecting between sample and holder. Below we describe a simple procedure (see Fig. 3 ▸) in which the angular information can be recovered in such a case. This correction is advisable, as the precision of the *s factor* (see Section 2.3) is affected by imprecise refraction parameters.

The refinement of the refraction parameters should proceed along the following steps:

(i) Refine the incident angle so that the diffuse tail of the reflected beam close to the beamstop comes out correctly.

(ii) If the position of the reflected beam is not clear, adjust the incident angle to get the sample horizon (see Fig. 3 ▸, indicated by the yellow line) and the lower edge of the Yoneda band right.

(iii) The critical angle of the substrate can be obtained from the CXRO online calculator (CXRO, 2021 ▸) knowing the beam energy.

(iv) Then refine the film critical angle by fitting the intensity maximum at the lower edge of the Yoneda band.

At this point the refraction parameters are established (*alf_i*, *alp_cF*, *alf_cS*).

### 3.3. In-plane lattice

Next, the lattice constants in the surface plane are to be established. These are not affected by refraction or dynamic effects but are the result of 2D powder scattering. So, for instance, a hexagonal in-plane structure would yield the tell-tale series 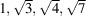,…, if all parallel components of the reflections are taken into account. If there is already an expectation with regards to the lattice type (*fcc* or *hcp*), one can start from there with some trial and error to fit the in-plane lattice. Otherwise one can use a simple hexagonal lattice as a starting point to establish the in-plane lattice constant. For lattices of low symmetry, such as GIWAXS of molecular thin films, a known bulk structure of the molecule or a related molecule can provide a good starting point, or the in-plane lattice can be fitted, as detailed in Sections 3.5–3.7.

### 3.4. Out-of-plane lattice and shrinkage factor

Again the example of the in-plane hexagonal lattice is quite revealing on how to proceed: an in-plane hexagonal lattice can be due to *fcc* (111) and *hcp* (001), but also simple hexagonal, such as for standing cylinders in a block copolymer; even *bcc* (110) appears as a slightly distorted hexagonal lattice in-plane. The next indicator of the lattice symmetry is the stacking. For instance, *fcc* (111) features ABC stacking; hence there are two reflections between the diffuse projections of the main series reflections close to the beamstop. Both *hcp* (001) and *bcc* (110) feature AB stacking, and thus there is only one reflection in between main series reflections. For simple hexagonal or cubic lattices with (001) orientation all reflections are in lockstep in the vertical direction corresponding to simple AA stacking. Even if the reflections close to the beamstop are not clear or not visible, the sequence of reflection positions along the vertical direction will indicate the stacking [see Smilgies (2025 ▸)]. Ultimately the pattern of allowed and forbidden reflections that the program calculates according to the Bravais type of the lattice and the index rules will determine the lattice symmetry and orientation relative to the substrate.

Low-symmetry lattices of molecules tend to be much harder to index. Starting from a known bulk structure can provide a good starting point. The parallel plane orientation can be established if there is a θ–2θ scan available, or if molecular visualization software such as *Mercury* (Macrae *et al.*, 2020 ▸) from the Cambridge Crystallographic Data Centre can help to identify close-packed planes. Molecular lattices feature a large range of scattering intensities, and it is not always clear whether a missing spot is due to a weak reflection or space-group symmetry. The *indexGIXS* program provides additional space-group elements to use with the Bravais lattices, such as glide planes and screw axes, which help to determine whether systematic absences do occur. These rules can be set using the menu invoked by the *index rule* pushbutton.

**Figure 4:**
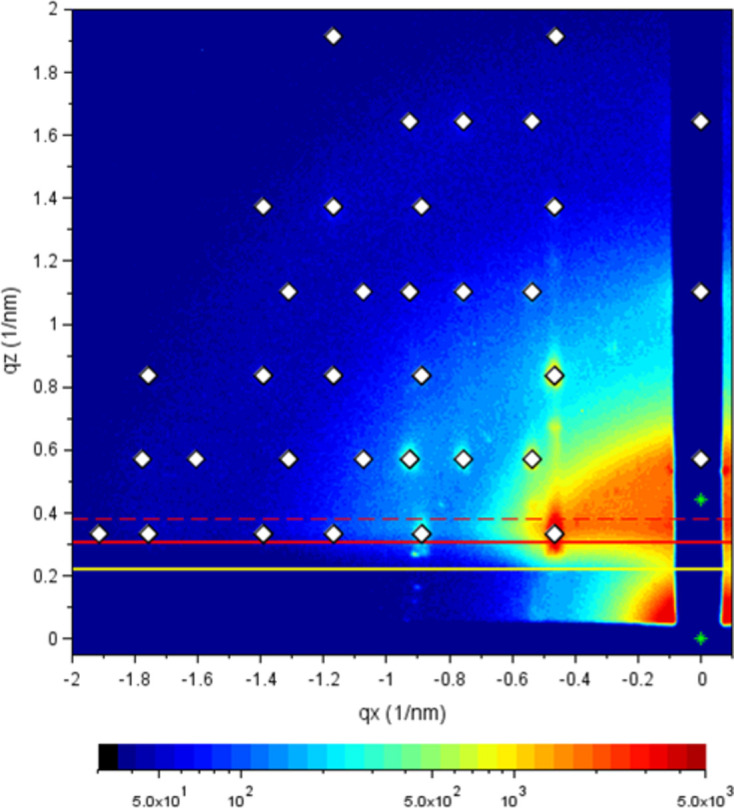
Indexing a *bcc* (110) superlattice of octahedral Pt_2_Cu_3_ nanoparticles. Publication-style *indexGIXS* output was generated for the SAXS regime.

The final step is to refine the shrinkage/swelling parameter so that the perpendicular spot positions are matched. The accuracy of this step is determined by having resolved the refraction parameters correctly. As refraction effects are more pronounced close to the Yoneda band, one should start fitting the reflection at higher exit angles. If discrepancies remain, this is often an indication that the beam position or the refraction parameters need additional fine tuning. If all falls into place, as shown in Fig. 4 ▸, the full structure including lattice type and parameters, parallel plane, and shrinkage parameter are established, along with the critical angles of the film.

### 3.5. Low-symmetry lattices

As an example of a more elaborate application we show the indexing of a wide-angle scattering pattern of a thin film of pyrene deposited on a glass slide. In this case the G2 ψ-axis diffractometer was used for reciprocal-space mapping using a linear diode array as detector (Smilgies *et al.*, 2005 ▸; Nowak *et al.*, 2006 ▸). The ν angle was scanned around the vertical axis while the δ angle was determined by the calibration of the linear detector. At room temperature pyrene crystallizes as polymorph I in the monoclinic space group *P*2_1_/*a* (Camerman & Trotter, 1965 ▸; Kai *et al.*, 1978 ▸). Hence, in the *index rule* menu the unit cell was chosen as primitive (*P*), and, in addition, the index rules for the *a* glide plane (denoted ‘*b*/*a*’) and the twofold screw axis along the *b* direction were selected via the *index rule* pushbutton. The label of the pushbutton is changed to a red color in order to indicate that a space-group element was invoked.

**Figure 5:**
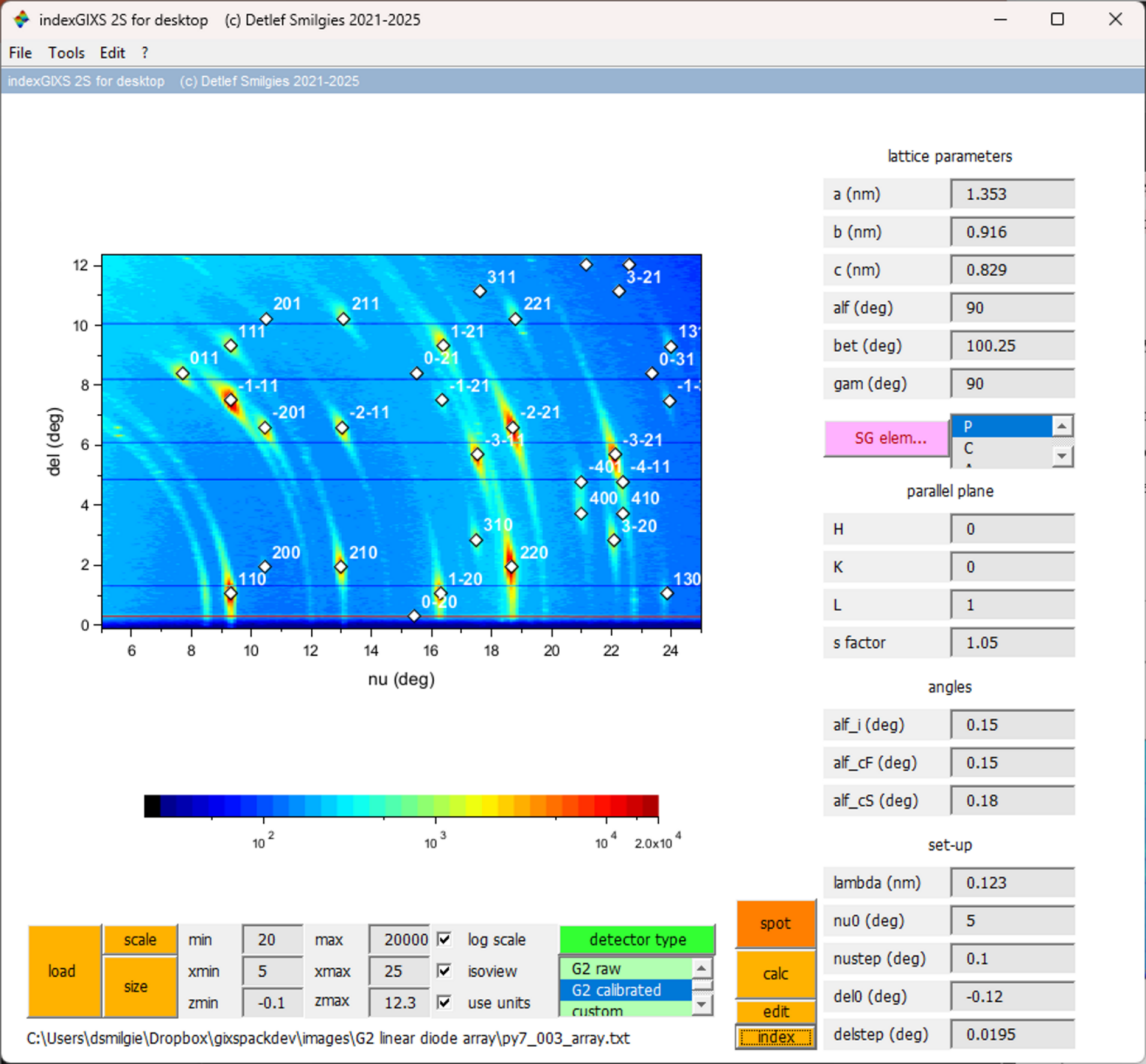
Indexing the monoclinic structure of pyrene from a reciprocal space map obtained with a linear diode array mounted on a ψ-circle diffractometer. The dark horizontal lines are due to dead elements in the diode array. The successful indexing with a known bulk polymorph shows that pyrene assumes the room-temperature bulk structure with the (001) plane parallel to the substrate surface. A 5% swelling was included for the dip-coated molecular film. Reproduced from Smilgies (2025 ▸) under the terms of the Creative Commons Attribution (CC BY) license (https://creativecommons.org/licenses/by/4.0/).

After lattice parameters, space group and instrument parameters were set, it could be established that the pyrene thin film crystallized in polymorph I with the (001) plane parallel to the substrate. This being a known bulk polymorph, the known crystal structure can be used to establish how the molecules are oriented with regard to the substrate surface. In addition we found a 5% swelling of the thin film deposited by dip coating, as compared with the bulk structure. The final indexing is shown in Fig. 5 ▸. Other known pyrene polymorphs occur below 110 K and under pressure (Zhou *et al.*, 2024 ▸); however, these could be excluded.

### 3.6. Indexing in reciprocal space

**Figure 6:**
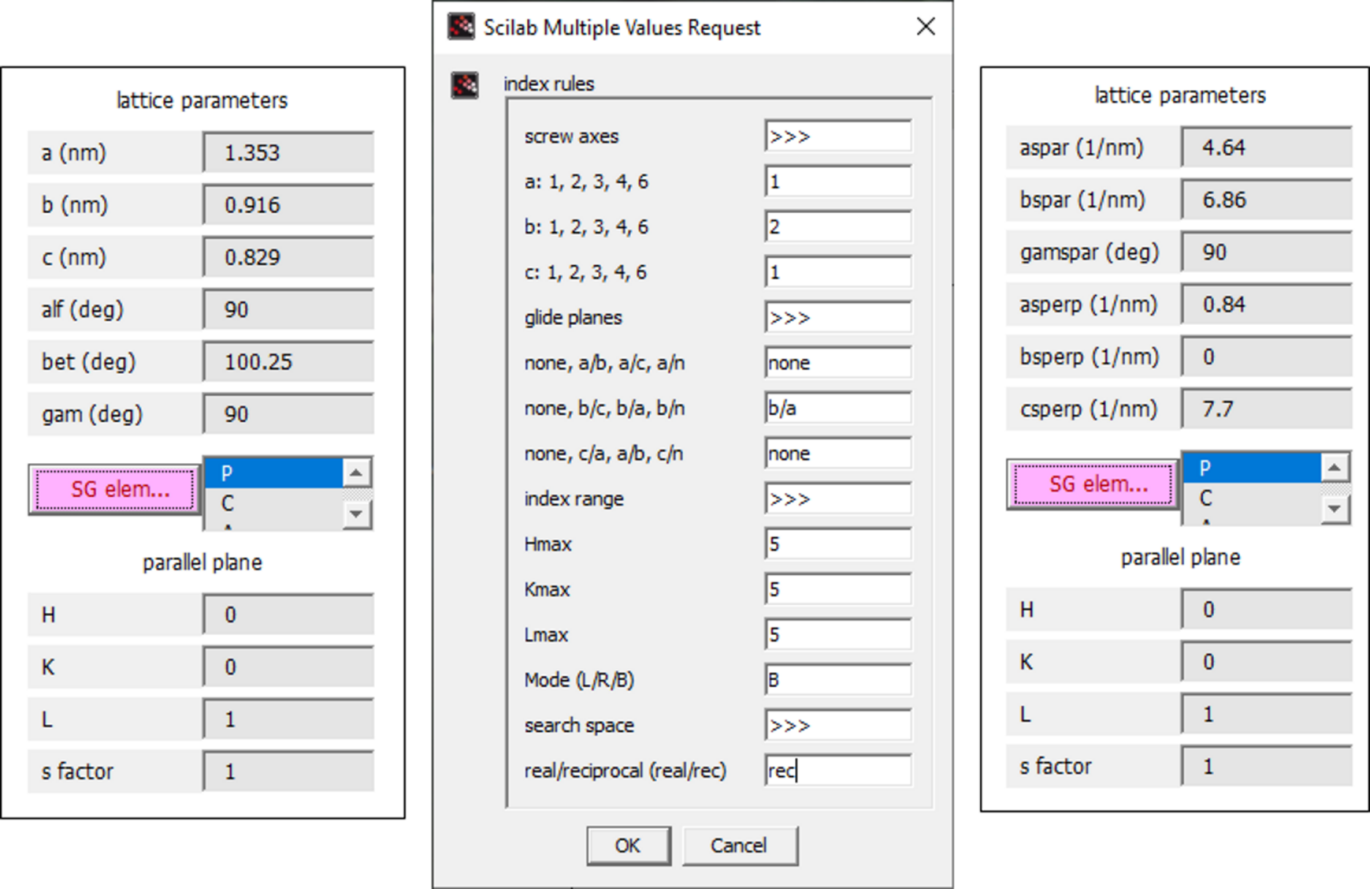
Switching from the real-space surface unit cell (left) to the reciprocal surface unit cell (right) using the *index rule/SG element* menu (middle), while maintaining the *P*2_1_/*a* index rule.

Structures with low symmetry such as triclinic and monoclinic are notoriously difficult to index if there is no closely matching known bulk structure. Starting with *indexGIXS* version 2S there is an option in the *index rule* menu to index in reciprocal space. This option is restricted to structures using the surface unit cell, *i.e.* when *HKL* = 001. Such a surface unit cell can be found for any 2D powder (Smilgies, 2026 ▸). When using the surface unit cell or an equivalent bulk unit cell, the reciprocal lattice vector *cs* is perpendicular to the surface plane, *i.e.* its parallel component equals 0. On the other hand, the reciprocal lattice vectors *as* and *bs* have parallel components, *aspar* and *bspar*, and also perpendicular components, *asperp* and *bsperp*, because the angles of *as* and *bs* with *cs* are in general different from 90°. Hence the surface reciprocal lattice parameters can be rewritten as in-plane components *aspar*, *bspar* and the enclosed angle *gamspar*, as well as the normal components *asperp*, *bsperp* and *csperp* (see Fig. 6 ▸). The advantage of this representation is that parallel and perpendicular lattice parameters are now decoupled, *i.e.* changes in *aspar*, *bspar* and *gamspar* cause a shift of reflections in 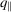 while changes in *asperp*, *bsperp* or *csperp* move reflections along 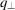. Here, 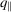 and 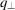 are the parallel and perpendicular components of the scattering vector, respectively, relative to the reference plane.

**Figure 7:**
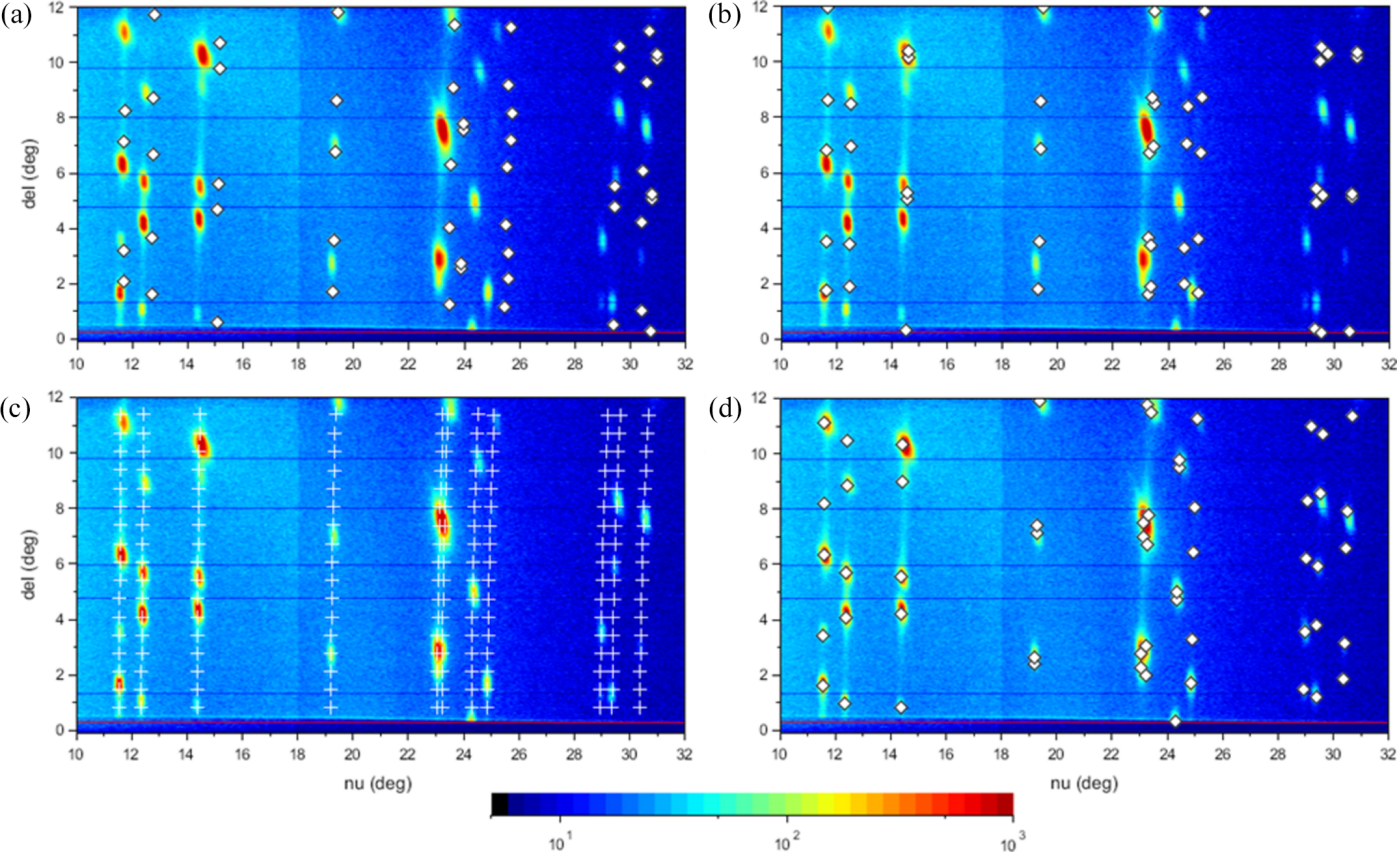
Indexing a TES-ADT thin film. (*a*) α phase and (*b*) δ phase from previous work. (*c*) Indexing in reciprocal space: tracing the scattering rods to find the in-plane structure by setting *csperp* to an arbitrary small value (here, 0.5 nm^−1^). (*d*) With a good match of the in-plane structure, *asperp* and *bsperp* were fitted to the lowest *q*_*z*_ values of the (10*l*) and (01*l*) rods identified in the first step. Finally, *csperp* was adjusted to match the reflections along the rods.

The indexing strategy in reciprocal space is to try to first get a good guess for the in-plane lattice. This is done by assuming the lowest-order reflections in 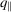 to be (10*l*) and the second lowest reflection to be (01*l*), and then matching up *aspar* and *bspar*. In thin-film and surface scattering it is useful to group reflections together with the same *hk* values and a variety of *l* values – the ‘scattering rods’. At this step it is helpful to choose an arbitrary small value for *csperp* for tracing the whole scattering rods [see Fig. 7 ▸(*c*)]. Finally, the third-order rod is assumed to be (11*l*) as a trial and its location is matched by adjusting *gamspar*. The deeper reasoning and further refinement of this ansatz are described by Smilgies (2026 ▸).

If this trial looks promising, as shown for the case of a thermally annealed thin film of bis­(tri­ethyl­silylethynyl) anthradi­thio­phene (TES-ADT) molecules used here as a demonstration, then the out-of-plane structure can be tackled. Initial values for *asperp* and *bsperp* can be found by fitting the reflections of the (10*l*) and (01*l*) rods closest to the sample horizon. For this purpose, *csperp* can be temporarily set to a large value. Then *csperp* is adjusted to provide an appropriate spacing of reflections along the rods [Fig. 7 ▸(*d*)]. Finally, small tweaks of the parameters can be used for further improvement and then the reciprocal lattice parameters can be back-transformed into the conventional real-space lattice constants. For comparison, the calculated reflection positions for the α and δ phases from the literature (Yu *et al.*, 2021 ▸) are provided [Figs. 7 ▸(*a*) and 7 ▸(*b*), respectively].

A recent review lists four polymorphs of TES-ADT (Yu *et al.*, 2021 ▸) on the basis of earlier studies (Payne *et al.*, 2005 ▸; Lee *et al.*, 2012 ▸; Chen *et al.*, 2014 ▸). While the α and δ polymorphs are close to the thin-film phase explained above, the set of lattice parameters found here fits the scattering pattern considerably better. A comparison of the three structures is shown in Table 1 ▸ and in Fig. 7 ▸.

While the outlined procedure worked extremely well in the present case, there can be various complications, such as space-group-forbidden scattering rods. However, recently it was shown that the reciprocal surface unit cell can be reconstructed on the basis of the in-plane **q** vectors of the first few scattering rods (Smilgies, 2026 ▸). This systematic method works very efficiently if combined with *indexGIXS* for visualization. After the in-plane lattice parameters *aspar*, *bspar* and *gamspar* are determined, the vertical reciprocal lattice vector components *asperp*, *bsperp* and *csperp* can be easily identified from the (10*l*), (01*l*),and (00*l*) scattering rods, respectively. By back-transforming the reciprocal lattice vectors using the *real/rec* parameter in the *index rule* menu (see Fig. 6 ▸), the real-space surface unit cell can be obtained, as shown in Table 1 ▸.

### 3.7. Equivalent choices of lattice parameters

**Figure 8:**
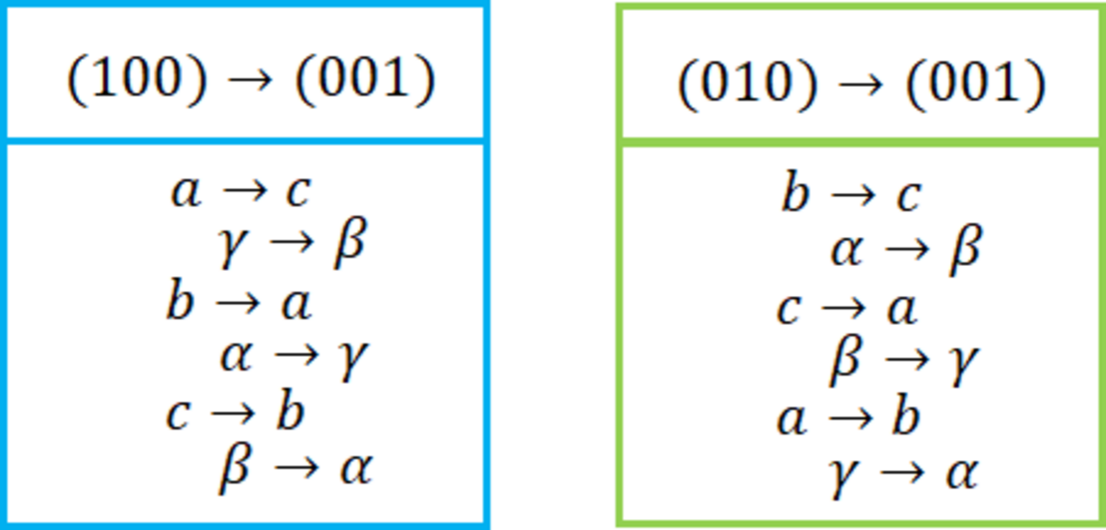
Cyclic permutations of triclinic lattice parameters to map the single-crystal lattice parameters onto the thin-film surface unit cell.

It may transpire for triclinic lattices that an (*HKL*) plane such as (100) or (010) with regard to the bulk lattice parameters provides a convenient starting point for indexing. However, for indexing in reciprocal space, a surface unit cell with (*HKL*) equal to (001) is needed. In general, a specific choice of lattice parameters is not unique in crystallography. In particular, cyclic permutation of *a*, *b* and *c* as well as of *alf*, *bet* and *gam* results in an equivalent description of the triclinic lattice. Fig. 8 ▸ shows in a simple map how bulk lattice parameters can be reshuffled to yield a (001) reference plane (*HKL*) and thus a surface unit cell.

**Figure 9:**
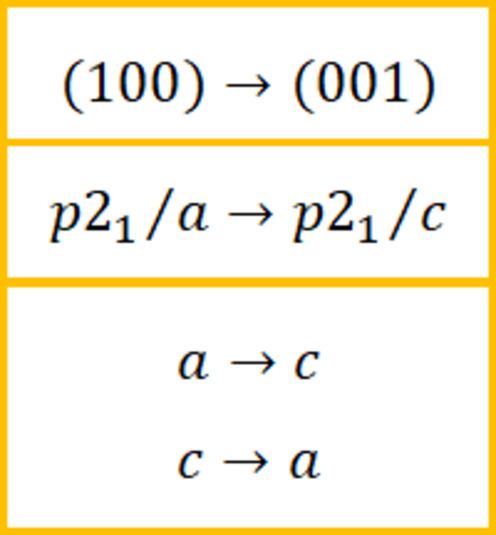
Mapping monoclinic unit-cell parameters and space group to yield a surface unit cell. Lattice parameters 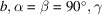 remain unchanged.

A similar procedure can be applied for monoclinic lattices. However, for the monoclinic space groups with a glide plane some care is needed. Here we discuss space group *P*2_1_/*c* with a (100) *HKL* plane as found for instance for perylene (Smilgies, 2025 ▸). The task is now to find a new set of lattice parameters while maintaining the glide-plane symmetry. Since the special direction *b* is perpendicular to the plane spanned by *a* and *c*, we can focus on this plane and use the simple transformation shown in Fig. 9 ▸.

The *b* direction and all angles remain the same. It can be easily verified with *indexGIXS* that the new unit cell indeed yields the same reflection pattern.

## 4. Usage, software requirements and distribution

The *indexGIXS* program has been successfully applied by a number of user groups apart from ourselves to determine lattice type, polymorph and lattice orientation in molecular and nanostructured thin films. The easy-to-use user interface displays all relevant parameters in a logical arrangement, multiple detector formats are supported, and indexing tools are provided in real and reciprocal space. The *indexGIXS* program is also a useful tool to familiarize the user with the intricacies of indexing 2D grazing-incidence scattering patterns.

Recently more sophisticated computer-based approaches have emerged that automate thin-film structure determination [as reported, for instance, by Mannsfeld *et al.* (2011 ▸), Hailey *et al.* (2014 ▸), Savikhin *et al.* (2020 ▸) and Kainz *et al.* (2021 ▸)]. However, a recent study found that, as long as the first few in-plane *q* values can be derived from the observed scattering rods, there is a simple systematic procedure to obtain *aspar*, *bspar* and *gamspar* (Smilgies, 2026 ▸) from which the full reciprocal surface unit cell and then the usual real-space lattice parameters can be determined, as outlined in Fig. 7 ▸.

Regardless of the method, a refinement process is often necessary to improve an initially identified lattice parameter set, and *indexGIXS* is well equipped for providing such a refinement. Refinement in reciprocal space is particularly useful to improve matching of high-order reflections, which require subtle tweaks of lattice parameters that leave the low-order reflections practically unchanged but can have significant effect on high-order reflections. After refining the surface unit cell of the film in reciprocal space the *index rule* menu can be used again to back-transform to real space and obtain a refined set of standard real-space lattice parameters (Smilgies, 2026 ▸).

The *indexGIXS* program uses the current *Scilab 2026.0.0* distribution compatible with MS Windows, Linux and MacOS platforms. The *Scilab* software and development environment can be downloaded free of charge from https://www.scilab.org. The *indexGIXS* program is part of a suite of programs for the analysis of area-detector data for grazing-incidence scattering, which is freely distributed by the corresponding author on request. The latest version of *indexGIXS* is available on Github as well: https://github.com/smilgies/scilab/tree/main/indexGIXS-3A.

## Figures and Tables

**Table 1:** Comparison of lattice parameters of TES-ADT polymorphs close to the thin-film phase published by Yu *et al.* (2021 ▸)

Polymorph	*a* (nm)	*b* (nm)	*c* (nm)	α (°)	β (°)	γ (°)
α	0.67	0.73	1.67	98.1	94.5	103.9
δ	0.69	0.74	1.66	96	96	106
This work	0.70	0.75	1.76	96.37	91.70	106.35

## References

[bb1] Bian, K., Choi, J. J., Kaushik, A., Clancy, P., Smilgies, D.-M. & Hanrath, T. (2011). *ACS Nano* **5**, 2815–2823. 10.1021/nn103303q 21344877

[bb2] Breiby, D. W., Bunk, O., Andreasen, J. W., Lemke, H. T. & Nielsen, M. M. (2008). *J. Appl. Cryst.* **41**, 262–271.

[bb3] Busch, P., Rauscher, M., Smilgies, D.-M., Posselt, D. & Papadakis, C. M. (2006). *J. Appl. Cryst.* **39**, 433–442.

[bb4] Camerman, A. & Trotter, J. (1965). *Acta Cryst.* **18**, 636–643.

[bb5] Chavis, M. A., Smilgies, D.-M., Wiesner, U. & Ober, C. K. (2015). *Adv. Funct. Mater.* **25**, 3057–3065. 10.1002/adfm.201404053 PMC4724432 26819574

[bb6] Chen, J., Shao, M., Xiao, K., Rondinone, A. J., Loo, Y.-L., Kent, P. R. C., Sumpter, B. G., Li, D., Keum, J. K., Diemer, P. J., Anthony, J. E., Jurchescu, O. D. & Huang, J. (2014). *Nanoscale* **6**, 449–456. 10.1039/c3nr04341j 24217182

[bb7] Crossland, E., Kamperman, M., Nedelcu, M., Ducati, C., Wiesner, U., Smilgies, D., Toombes, G. E. S., Hillmyer, M. A., Ludwigs, S., Steiner, U. & Snaith, H. (2009). *Nano Lett.* **9**, 2807–2812. 10.1021/nl803174p 19007289

[bb30] CXRO (2021). *X-ray Interactions With Matter*, https://henke.lbl.gov/optical_constants/.

[bb8] Hailey, A. K., Hiszpanski, A. M., Smilgies, D.-M. & Loo, Y.-L. (2014). *J. Appl. Cryst.* **47**, 2090–2099. 10.1107/S1600576714022006 PMC4248569 25484845

[bb9] Jiang, Z. (2015). *J. Appl. Cryst.* **48**, 917–926.

[bb10] Jiang, Z., Lee, D. R., Narayanan, S., Wang, J. & Sinha, S. K. (2011). *Phys. Rev. B* **84**, 075440.

[bb11] Kai, Y., Hama, F., Yasuoka, N. & Kasai, N. (1978). *Acta Cryst.* B**34**, 1263–1270.

[bb12] Kainz, M. P., Legenstein, L., Holzer, V., Hofer, S., Kaltenegger, M., Resel, R. & Simbrunner, J. (2021). *J. Appl. Cryst.* **54**, 1256–1267. 10.1107/S1600576721006609 PMC8366425 34429726

[bb13] Lee, B., Park, I., Yoon, J., Park, S., Kim, J., Kim, K.-W., Chang, T. & Ree, M. (2005). *Macromolecules* **38**, 4311–4323.

[bb14] Lee, S. S., Tang, S. B., Smilgies, D.-M., Woll, A. R., Loth, M. A., Mativetsky, J. M., Anthony, J. E. & Loo, Y.-L. (2012). *Adv. Mater.* **24**, 2692–2698. 10.1002/adma.201104619 22511330

[bb40] Macrae, C. F., Sovago, I., Cottrell, S. J., Galek, P. T. A., McCabe, P., Pidcock, E., Platings, M., Shields, G. P., Stevens, J. S., Towler, M. & Wood, P. A. (2020). *J. Appl. Cryst.* **53**, 226–235. 10.1107/S1600576719014092 PMC6998782 32047413

[bb15] Mannsfeld, S. C. B., Tang, M. L. & Bao, Z. (2011). *Adv. Mater.* **23**, 127–131. 10.1002/adma.201003135 21104808

[bb16] Nowak, D. E., Blasini, D. R., Vodnick, A. M., Blank, B., Tate, M. W., Deyhim, A., Smilgies, D.-M., Abruña, H., Gruner, S. M. & Baker, S. P. (2006). *Rev. Sci. Instrum.* **77**, 113301.

[bb17] Paik, M. Y., Bosworth, J. K., Smilges, D. M., Schwartz, E. L., Andre, X. & Ober, C. K. (2010). *Macromolecules* **43**, 4253–4260. 10.1021/ma902646t PMC2992440 21116459

[bb18] Payne, M. M., Parkin, S. R., Anthony, J. E., Kuo, C. & Jackson, T. N. (2005). *J. Am. Chem. Soc.* **127**, 4986–4987. 10.1021/ja042353u 15810810

[bb19] Posselt, D., Zhang, J., Smilgies, D.-M., Berezkin, A., Potemkin, I. I. & Papadakis, C. M. (2017). *Prog. Polym. Sci.* **66**, 80–115.

[bb20] Savikhin, V., Steinrück, H.-G., Liang, R.-Z., Collins, B. A., Oosterhout, S. D., Beaujuge, P. M. & Toney, M. F. (2020). *J. Appl. Cryst.* **53**, 1108–1129.

[bb22] Smilgies, D.-M. (2019). *J. Appl. Cryst.* **52**, 247–251.

[bb23] Smilgies, D.-M. (2022). *J. Polym. Sci.* **60**, 1023–1041.

[bb24] Smilgies, D.-M. (2025). *Crystals* **15**, 63.

[bb25] Smilgies, D.-M. (2026). *Crystals* **16**, 43.

[bb26] Smilgies, D.-M. & Blasini, D. R. (2007). *J. Appl. Cryst.* **40**, 716–718.

[bb27] Smilgies, D.-M., Blasini, D. R., Hotta, S. & Yanagi, H. (2005). *J. Synchrotron Rad.* **12**, 807–811. 10.1107/S0909049505030815 16239752

[bb28] Stein, G. E., Kramer, E. J., Li, X. & Wang, J. (2007). *Macromolecules* **40**, 2453–2460.

[bb29] Tate, M. P., Urade, V. N., Kowalski, J. D., Wei, T.-C., Hamilton, B. D., Eggiman, B. W. & Hillhouse, H. W. (2006). *J. Phys. Chem. B* **110**, 9882–9892. 10.1021/jp0566008 16706443

[bb31] Weidman, M. C., Smilgies, D.-M. & Tisdale, W. A. (2016). *Nat. Mater.* **15**, 775–781. 10.1038/nmat4600 26998914

[bb32] Yu, L., Portale, G. & Stingelin, N. (2021). *J. Mater. Chem. C.* **9**, 10547–10556.

[bb33] Zhang, J., Luo, Z., Martens, B., Quan, Z., Kumbhar, A., Porter, N., Wang, Y., Smilgies, D.-M. & Fang, J. (2012). *J. Am. Chem. Soc.* **134**, 14043–14049. 10.1021/ja304108n 22839450

[bb34] Zhou, W., Yin, Y., Laniel, D., Aslandukov, A., Bykova, E., Pakhomova, A., Hanfland, M., Poreba, T., Mezouar, M., Dubrovinsky, L. & Dubrovinskaia, N. (2024). *Commun. Chem.* **7**, 209. 10.1038/s42004-024-01294-0 PMC11405754 39285188

